# Depression, anxiety and brain volume after hearing loss and tinnitus: cohort study in the UK Biobank

**DOI:** 10.1192/bjo.2023.634

**Published:** 2024-02-01

**Authors:** Xiaowan Chen, Kejia Hu, Huan Song, Li Yin, Magnus Kaijser, Tiril P. Gurholt, Ole A. Andreassen, Unnur Valdimarsdóttir, Fang Fang, Maoli Duan

**Affiliations:** Department of Otolaryngology Head and Neck Surgery, the First Hospital of Lanzhou University, Lanzhou, Gansu Province, China; Unit of Integrative Epidemiology, Institute of Environmental Medicine, Karolinska Institutet, Stockholm, Sweden; Department of Otolaryngology Head and Neck Surgery & Audiology and Neurotology, Karolinska University Hospital, Stockholm, Sweden; and Department of Clinical Science, Intervention and Technology, Karolinska Institutet, Stockholm, Sweden; Unit of Integrative Epidemiology, Institute of Environmental Medicine, Karolinska Institutet, Stockholm, Sweden; West China Biomedical Big Data Center, West China Hospital, Sichuan University, Chengdu, China; Med-X Center for Informatics, Sichuan University, Chengdu, China; and Centre of Public Health Sciences, Faculty of Medicine, University of Iceland, Reykjavik, Iceland; Department of Medical Epidemiology and Biostatistics, Karolinska Institutet, Stockholm, Sweden; Unit of Integrative Epidemiology, Institute of Environmental Medicine, Karolinska Institutet, Stockholm, Sweden; and Department of Neuroradiology, Karolinska University Hospital, Stockholm, Sweden; Norwegian Centre for Mental Disorders Research (NORMENT), Division of Mental Health and Addiction, Oslo University Hospital, Oslo, Norway; and Institute of Clinical Medicine, University of Oslo, Oslo, Norway; Unit of Integrative Epidemiology, Institute of Environmental Medicine, Karolinska Institutet, Stockholm, Sweden; Centre of Public Health Sciences, Faculty of Medicine, University of Iceland, Reykjavik, Iceland; and Department of Epidemiology, Harvard T.H. Chan School of Public Health, Boston, Massachusetts, USA; Department of Otolaryngology Head and Neck Surgery & Audiology and Neurotology, Karolinska University Hospital, Stockholm, Sweden; and Department of Clinical Science, Intervention and Technology, Karolinska Institutet, Stockholm, Sweden

**Keywords:** Hearing loss, tinnitus, mood disorders, brain volume

## Abstract

**Background:**

Hearing loss and tinnitus have been proposed as potential indicators of impaired mental health and brain morphological changes.

**Aims:**

To assess the associations of hearing loss and tinnitus with the risk of depression and anxiety and with brain volume.

**Method:**

We conducted a community-based cohort study including 129 610 participants aged 40−69 years at recruitment to the UK Biobank with a follow-up period during 2006–2021 to estimate the risk of depression and anxiety after detection of hearing loss and reported tinnitus. We also assessed the associations of hearing loss and tinnitus with brain volume in a subsample with available brain magnetic resonance imaging data (*N* = 5222).

**Results:**

We observed an increased risk of depression among individuals with hearing loss (hazard ratio [HR] 1.14, 95% CI 1.03–1.26), tinnitus (HR 1.30, 95% CI 1.21–1.41) or both (HR 1.32, 95% CI 1.15–1.52), compared with individuals with neither hearing loss nor tinnitus. Similar results were noted for anxiety (HR 1.18, 95% CI 1.07–1.30 for hearing loss; HR 1.32, 95% CI 1.22–1.43 for tinnitus; and HR 1.48, 95% CI 1.30–1.68 for both). Hearing loss was associated with decreased overall brain volume as well as decreased volume of different brain regions. The latter associations disappeared after adjustment for whole intracranial volume. Tinnitus was associated with greater left accumbens and right occipital pole volume after adjustment for the whole intracranial volume.

**Conclusions:**

Individuals with tinnitus are at increased risk of depression and anxiety. Hearing loss, on the other hand, is associated with both mood disorders and altered brain morphology.

Hearing loss and tinnitus are common health problems in modern society and significantly affect the quality of life of those affected.^1,2^ The prevalence of hearing loss has been reported to vary between 11% and 20% in the general population and increases with age.^3^ The prevalence of tinnitus is reported to be between 8% and 25.3% and also increases with age.^4^ Hearing loss and tinnitus have both been suggested to be risk factors or indicators of poor mental health, including increased risk of psychiatric disorders.^5–8^ For instance, Brewster et al reported that self-reported hearing loss predicted depression over a 10 year follow-up,^6^ whereas Cejas et al found that adolescents with severe to profound hearing loss had higher rates of depression and anxiety.^7^ Oosterloo et al found more severe tinnitus to be associated with an increase in subsequent anxiety symptoms and poor sleep quality.^8^ Further, as neuroimaging studies have shown brain morphological alterations in relation to hearing loss and tinnitus in auditory regions and limbic system,^9–12^ hearing loss and tinnitus have also been proposed as modifiable potential early markers of neurological diseases and neuro-morphological changes.

Hearing loss and tinnitus frequently present simultaneously.^4,8^ However, previous studies have not always disentangled the effects of hearing loss from those of tinnitus, and *vice versa*, or analysed the impact of hearing loss and tinnitus by severity. To this end, we used UK Biobank data to investigate the associations of hearing loss and tinnitus with the risk of depression and anxiety and with brain volume, hypothesising that hearing loss and tinnitus would both be associated with higher risk of mood disorders as well as higher risk of neuro-morphological changes. We examined separately individuals with only hearing loss, those with only tinnitus and those with both, as well as individuals with different severity of hearing loss and tinnitus, to aid the identification of a high-risk population.

## Method

### Population

The UK Biobank (https://www.ukbiobank.ac.uk/) is a cohort study involving over 500 000 participants aged 40–69 who were recruited between 2006 and 2010. At recruitment, all participants were invited to 22 assessment centres in the UK, where they provided written informed consent and completed a touchscreen questionnaire, verbal interview, physical examination and biological sample collection. During 2012–2013, approximately 20 000 of these participants underwent the first repeated assessment. During 2014–2019, a subset of participants participated in the ongoing second and third repeat assessments, which included brain magnetic resonance imaging (MRI) examination. Image data capture and processing have been described in detail elsewhere (https://biobank.ndph.ox.ac.uk/).

**Fig. 1:**
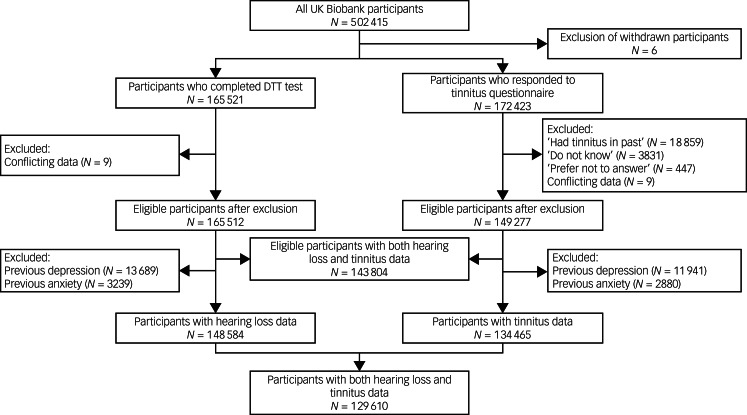
Flow chart of study population selection. DTT, digit triplet test.

The present study was a cohort study based on the UK Biobank, including all participants with available information on hearing ability and tinnitus at recruitment (*N* = 143 804) (Fig. 1). As we were primarily interested in new-onset depression and anxiety, we excluded participants with a previous diagnosis of depression or anxiety at recruitment, leaving 129 610 in the final cohort. We then followed these participants from the date of recruitment until the first diagnosis of depression (or anxiety), loss to follow-up, death or 30 September 2021, whichever came first. Participants who withdrew their informed consent were excluded (opt-out list dated 22 May 2022).

The authors assert that all procedures contributing to this work comply with the ethical standards of the relevant national and institutional committees on human experimentation and with the Helsinki Declaration of 1975, as revised in 2008. All procedures involving human subjects were approved by the NHS National Research Ethics Service (reference number: 16/NW/0274). We applied and obtained access to the UK Biobank data through application number 76 517. This study was also approved by the Swedish Ethical Review Authority (DNR: 2022-01516-01).

### Definitions of hearing loss and tinnitus

Hearing ability was evaluated by the digit triplet test (DTT) (Supplementary Methods available at https://doi.org/10.1192/bjo.2023.634), which is used to estimate hearing ability in noise.^13^ Based on the speech reception threshold (SRT), hearing loss was categorised as ‘yes’ (SRT ≥ −5.5 dB) or ‘no’ (SRT < −5.5 dB).^14^ Tinnitus was determined according to the question ‘Do you get or have you had noises (such as ringing or buzzing) in your head or in one or both ears that lasts for more than five minutes at a time?’ in the touchscreen questionnaire at recruitment and classified as ‘yes’ or ‘no’. According to hearing loss and tinnitus status at recruitment, we divided the cohort participants into the following categories: ‘tinnitus’, ‘hearing loss’, ‘both’ and ‘none’. In the secondary analyses, we further divided hearing loss as ‘insufficient hearing’ (SRT −5.5 to −3.5 dB) or ‘poor hearing’ (SRT > −3.5 dB) based on the result for the better-performing ear. We also divided tinnitus as ‘not bothersome’ or ‘bothersome’ based on the answer to the question ‘How much do these noises worry, annoy or upset you when they are at their worst?’ We included 134 456 participants who responded to the tinnitus questionnaire in the secondary analysis of severity of tinnitus, and 148 584 participants who completed the DTT test in the secondary analysis of severity of hearing loss (Fig. 1).

### Diagnoses of depression and anxiety

We ascertained diagnoses of depression and anxiety through health-related outcome data (Category 1712, https://biobank.ctsu.ox.ac.uk/ukb/label.cgi?id=1712) from the UK Biobank, according to ICD codes (Supplementary Table 1). Category 1712 includes complete in-patient hospital data since 1997 obtained through the Hospital Episode Statistics database in England, the Patient Database for Wales and the Scottish Mortality Record, as well as primary care data since 1990, covering about 45% of the UK Biobank participants, obtained through primary care computer system suppliers in England, Wales and Scotland.^15,16^ It also includes information from death registers and conditions self-reported at the assessment centres. We used ICD-10 codes F32 and F33 to ascertain depression and ICD-10 codes F40 and F41 to ascertain anxiety. The diagnoses of depression and anxiety in these data sources have been partly validated against detailed clinical evaluation, demonstrating a positive predictive value of over 80%.^17^

### Brain MRI

Using the available brain MRI data in the second repeat assessment from three dedicated centres, we selected eight brain regions of interest (ROIs) according to the literature.^9–12,18–21^ Subsequently, we studied three subcortical volumes (accumbens, amygdala and hippocampus), as well as five regional grey matter volumes (central opercular cortex, cingulate gyrus, hippocampus, inferior temporal gyrus and parahippocampal gyrus). To assess the specificity of the results, we also studied the whole intracranial volume and whole grey matter volume, as well as the volume of four brain regions with no relevance to hearing – the IX cerebellum (vermis), sensory cortex (postcentral gyrus), primary motor cortex (precentral gyrus) and vision cortex (occipital pole). We analysed the brain volumes in the left and right hemispheres separately as well as by subregion when relevant.

All data on brain volumes were obtained from T1-weighted MRI images by voxel-based morphometry, including FSL FAST for cortical structures (Category 1101), FSL FIRST for subcortical structures (Category 1102) and SIENAX analysis for total brain volume as previously shown.^22^ To reduce the possible impact of misclassification, in the analysis of brain volumes, we excluded participants who reported a different status of hearing loss and tinnitus between recruitment and second repeat assessment, leaving 4980 participants in the final analysis of brain volumes (43.2%).

### Covariates

Information on demographic and lifestyle factors including birth year, sex, education, annual household income and insomnia status was collected through a touchscreen questionnaire. Townsend deprivation score was calculated based on residential postcode at recruitment and used as a proxy of socioeconomic status. Information on psychiatric disorders (other than depression and anxiety) before recruitment was collected from health-related outcome data (Category 1712), according to ICD codes (Supplementary Table 1).

### Statistical analysis

We first described the baseline characteristics of the study participants, by hearing loss or tinnitus status. We used analysis of variance and chi-squared tests to determine the statistical significance of the differences among all four groups. We then used a univariable Cox model to assess the associations of these baseline characteristics, and of hearing loss and tinnitus, with the risk of depression or anxiety.

In the primary analysis, we used a multivariable Cox model to investigate the associations of hearing loss or tinnitus with the risk of depression and anxiety after adjusting for covariables shown to be statistically significantly associated with the risk of depression and anxiety in the univariable models; these included sex, age in years, educational attainment (college, tertiary, secondary or below secondary), income (>£100 000, £52 000–100 000, £31 000–51,999, £18 000–30 999 or <£18 000), insomnia status (usually, sometimes or never), Townsend index (by quartile distribution), history of other psychiatric disorders (yes or no) and assessment centre. To reduce the possibility of reverse causality, we used a 90 day lag time in all analyses. The proportional hazards assumption was examined using a test based on Schoenfeld residuals and found to hold. The results are presented as hazard ratios (HRs) with 95% confidence intervals. In addition to the Cox model, we used a flexible parametric model to explore changes in HR during the follow-up period, with the same adjustment and use of lag time as in the Cox model. To assess potential effect modification, we also performed stratified analyses by sex (male or female), previous psychiatric disorder (yes or no) and insomnia status (usually, sometimes or never). In the secondary analyses, we investigated the severity of hearing loss and tinnitus on the risk of depression and anxiety using the same models.

In the analysis of brain volumes, we first used a multivariable linear regression model to assess the associations of hearing loss, tinnitus or both with the different brain volumes, after adjustment of the same set of covariates as in the analyses of depression and anxiety. Second, to examine whether the associations for the selected ROIs were independent of whole brain volume, we further adjusted for the whole intracranial volume in the multivariable analyses of different ROIs.

Statistical analyses were conducted using R (version 4.0.2). Statistical significance was set at *P* < 0.05. All statistical tests were two-sided. Owing to multiple testing in the analyses of brain volume, the *P*-values in the multivariable linear regression were corrected by the Benjamini–Hochberg method. We considered a false discovery rate (FDR) of <0.05 to indicate statistical significance.

## Results

Of the 129 610 participants in the final cohort, 53.2% were female and 46.8% were male, with a mean (s.d.) age at recruitment of 57.4 (8.1) years (Table 1). A total of 11 975 participants (9.2%) had hearing loss, 20 359 (15.7%) reported tinnitus, and 4497 (3.5%) had both hearing loss and tinnitus at recruitment to the UK Biobank. Individuals with tinnitus or with both hearing loss and tinnitus were more likely to be male, compared with those with hearing loss alone (54.2% and 56.1% *v.* 45.2%). Presence of hearing loss or tinnitus, insomnia, higher Townsend index and previous psychiatric disorders were positively associated with the risk of depression and anxiety, whereas male sex, higher age, higher income and higher educational attainment were negatively associated with the risk of depression and anxiety (Supplementary Fig. 1).

**Table 1:** Baseline characteristicsof the 129 610 UK Biobank participants included in the present study, by hearing loss or tinnitus status

Characteristic	None (*N* = 92 779)	Only hearing loss (*N* = 11 975)	Only tinnitus (*N* = 20 359)	Both (*N* = 4497)	Total (*N* = 129 610)	*P*-value
Sex						<0.001
Female	51 085 (55.1)	6560 (54.8)	9327 (45.8)	1975 (43.9)	68 947 (53.2)	
Male	41 694 (44.9)	5415 (45.2)	11 032 (54.2)	2522 (56.1)	60 663 (46.8)	
Age at recruitment, years						<0.001
Mean (s.d.)	56.6 (8.2)	60.4 (7.5)	58.7 (7.6)	61.8 (6.8)	57.4 (8.1)	
Range	40.1–72.1	40.2–70.4	39.2–70.5	40.3–71.0	39.2–72.1	
Insomnia						<0.001
Usually	23 074 (24.9)	3110 (26.0)	6524 (32.0)	1461 (32.5)	34 169 (26.4)	
Sometimes	44 158 (47.6)	5693 (47.5)	9604 (47.2)	2141 (47.6)	61 596 (47.5)	
Never	25 492 (27.5)	3153 (26.3)	4211 (20.7)	887 (19.7)	33 743 (26.0)	
Unknown	55 (0.1)	19 (0.2)	20 (0.1)	8 (0.2)	102 (0.1)	
Educational attainment						<0.001
College	34 351 (37.0)	3567 (29.8)	6390 (31.4)	999 (22.2)	45 307 (35.0)	
Tertiary	30 721 (33.1)	3643 (30.4)	7114 (34.9)	1379 (30.7)	42 857 (33.1)	
Secondary	16 094 (17.3)	1932 (16.1)	3239 (15.9)	664 (14.8)	21 929 (16.9)	
No secondary	10 905 (11.8)	2627 (21.9)	3401 (16.7)	1367 (30.4)	18 300 (14.1)	
Unknown	708 (0.8)	206 (1.7)	215 (1.1)	88 (2.0)	1217 (0.9)	
Income (pounds)						<0.001
Greater than 100 000	5965 (6.4)	347 (2.9)	870 (4.3)	81 (1.8)	7263 (5.6)	
52 000 to 100 000	19 205 (20.7)	1327 (11.1)	3295 (16.2)	361 (8.0)	24 188 (18.7)	
31 000 to 51 999	22 124 (23.8)	2201 (18.4)	4522 (22.2)	699 (15.5)	29 546 (22.8)	
18 000 to 30 999	19 684 (21.2)	2823 (23.6)	4817 (23.7)	1019 (22.7)	28 343 (21.9)	
Less than 18 000	14 042 (15.1)	2969 (24.8)	4097 (20.1)	1431 (31.8)	22 539 (17.4)	
Unknown	11 759 (12.7)	2308 (19.3)	2758 (13.5)	906 (20.1)	17 731 (13.7)	
Townsend index						<0.001
High	31 408 (33.9)	4922 (41.1)	7057 (34.7)	1929 (42.9)	45 316 (35.0)	
Median	31 619 (34.1)	3785 (31.6)	6865 (33.7)	1405 (31.2)	43 674 (33.7)	
Low	29 610 (31.9)	3249 (27.1)	6413 (31.5)	1153 (25.6)	40 425 (31.2)	
Unknown	142 (0.2)	19 (0.2)	24 (0.1)	10 (0.2)	195 (0.2)	
Previous psychiatric disorders						<0.001
Yes	7699 (8.3)	942 (7.9)	1791 (8.8)	429 (9.5)	10 861 (8.4)	
No	85 080 (91.7)	11 033 (92.1)	18 568 (91.2)	4068 (90.5)	118 749 (91.6)	

### Risk of depression and anxiety

During a median follow-up of 7.8 years, 5502 participants of the cohort received a first-recorded diagnosis of depression, whereas 5224 received a first diagnosis of anxiety. Compared with individuals with neither hearing loss nor tinnitus, there was an increased risk of depression among individuals with hearing loss (HR 1.14; 95% CI 1.03–1.26), tinnitus (HR 1.30; 95% CI 1.21–1.41) or both (HR 1.32; 95% CI 1.15–1.52) (Table 2). Similar results were noted for anxiety among individuals with hearing loss (HR 1.18; 95% CI 1.07–1.30), tinnitus (HR 1.32; 95% CI 1.22–1.43) or both (HR 1.48; 95% CI 1.30–1.68). No clear pattern was noted in the stratified analyses by sex, previous psychiatric disorders or insomnia.

**Table 2:** Hazard ratio (HR) and 95% CI values for depression or anxiety in relation to hearing loss or tinnitus, using participants with neither hearing loss nor tinnitus as reference^a^

Group	HR (95% CI) of depression			HR (95% CI) of anxiety		
	Hearing loss	Tinnitus	Both	Hearing loss	Tinnitus	Both
Overall	1.14 (1.03–1.26)	1.30 (1.21–1.41)	1.32 (1.15–1.52)	1.18 (1.07–1.30)	1.32 (1.22–1.43)	1.48 (1.30–1.68)
Stratified by sex						
Male	1.08 (0.91–1.27)	1.25 (1.11–1.41)	1.29 (1.05–1.58)	1.19 (1.01–1.41)	1.29 (1.14–1.46)	1.41 (1.15–1.73)
Female	1.18 (1.04–1.34)	1.34 (1.21–1.49)	1.34 (1.11–1.62)	1.17 (1.04–1.32)	1.35 (1.22–1.48)	1.54 (1.30–1.82)
Stratified by previous psychiatric disorders						
Yes	1.09 (0.84–1.43)	1.39 (1.15–1.67)	1.61 (1.16–2.23)	0.95 (0.72–1.26)	1.15 (0.93–1.41)	1.49 (1.06–2.10)
No	1.15 (1.03–1.27)	1.29 (1.19–1.41)	1.27 (1.09–1.48)	1.21 (1.10–1.34)	1.36 (1.25–1.47)	1.48 (1.29–1.71)
Stratified by insomnia						
Usually	1.11 (0.94–1.31)	1.39 (1.23–1.56)	1.43 (1.16–1.75)	1.26 (1.07–1.48)	1.37 (1.22–1.55)	1.77 (1.46–2.15)
Sometimes	1.23 (1.07–1.42)	1.26 (1.12–1.42)	1.20 (0.97–1.49)	1.17 (1.02–1.35)	1.35 (1.21–1.52)	1.28 (1.04–1.57)
Never	0.98 (0.76–1.26)	1.11 (0.88–1.39)	1.42 (0.98–2.05)	1.05 (0.83–1.33)	1.07 (0.86–1.33)	1.27 (0.88–1.85)

a. Adjusted for age, sex, income, educational level, insomnia, Townsend index, previous psychiatric disorders and assessment centre. All analyses used a 90-day lag time.

In the secondary analyses, we found a dose–response relationship for both associations. The HRs for first-recorded diagnosis of depression were 1.08 (95% CI 0.99–1.17) for insufficient hearing and 1.34 (95% CI 1.14–1.57) for poor hearing (*P* for trend <0.001), whereas those for anxiety were 1.10 (95% CI 1.01–1.19) for insufficient hearing and 1.23 (95% CI 1.04–1.45) for poor hearing (*P* for trend = 0.001), compared with no hearing loss (Supplementary Table 2). The HRs for depression were 1.19 (95% CI 1.10–1.28) for not-bothersome tinnitus and 1.60 (95% CI 1.43–1.78) for bothersome tinnitus (*P* for trend <0.001), whereas those for anxiety were 1.21 (95% CI 1.12–1.30) for not-bothersome tinnitus and 1.71 (95% CI 1.53–1.90) for bothersome tinnitus (*P* for trend <0.001), compared with no tinnitus (Supplementary Table 3).

**Fig. 2:**
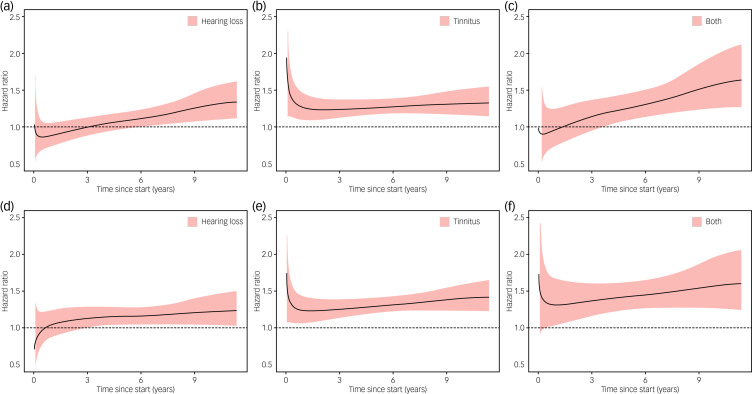
Changes in hazard ratios for depression (a–c) and anxiety (d–f) in relation to hearing loss or tinnitus during the follow-up period. A flexible parametric model was used to demonstrate the changes in hazard ratios during the follow-up period, with participants with neither hearing loss nor tinnitus as reference. All analyses used a 90-day lag time.

Figure 2 shows the temporal changes in HR for first-recorded diagnosis of depression and anxiety during follow-up among individuals with hearing loss, tinnitus or both, compared with individuals with neither. The association of hearing loss with depression or anxiety appeared to become stronger over time, whereas the association of tinnitus with depression or anxiety appeared to be relatively consistent over time. In the secondary analyses, individuals with poor hearing appeared to have a greater increase in risk of depression and anxiety, compared with individuals with insufficient hearing, although the difference was not statistically significant (Supplementary Fig. 2). By contrast, the increase in risk of depression and anxiety was constantly greater among individuals with bothersome tinnitus, compared with individuals with not-bothersome tinnitus, during follow-up.

### Brain MRI

**Fig. 3:**
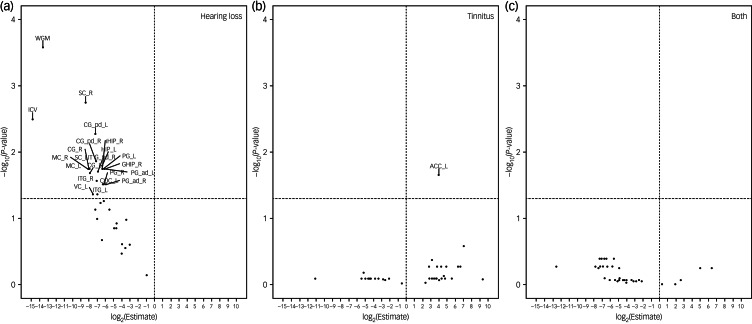
Brain volumes in relation to hearing loss or tinnitus. Volcano plots show brain volumes in relation to hearing loss or tinnitus based on a linear regression after adjustment for age, sex, income, educational level, insomnia, Townsend index and previous psychiatric disorders, with participants with neither hearing loss nor tinnitus as reference. Horizontal line denotes level of statistical significance with a false discovery rate of 0.05. The regions of interest include whole grey matter (WGM), intracranial volume (ICV), accumbens (ACC), amygdala (AMY), hippocampus (HIP), central opercular cortex (COC), cingulate gyrus (CG), grey matter hippocampus (GHIP), inferior temporal gyrus (ITG), parahippocampal gyrus (PG), postcentral gyrus (sensory cortex, SC), precentral gyrus (motor cortex, MC), occipital pole (vision cortex, VC) and cerebellum (CE). L, left; R, right.

**Fig. 4:**
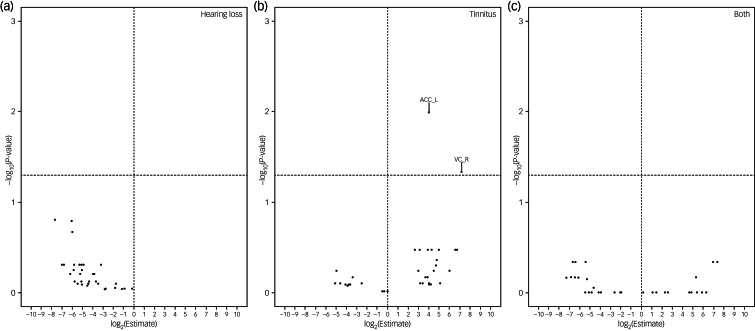
Brain volumes in relation to hearing loss or tinnitus after further adjustment for whole intracranial volume. Volcano plots show brain volumes in relation to hearing loss or tinnitus based on a linear regression after adjustment for intracranial volume, age, sex, income, educational level, insomnia, Townsend index and previous psychiatric disorders, with participants with neither hearing loss nor tinnitus as reference. Horizontal line denotes level of statistical significance with a false discovery rate of 0.05. ACC_L, left accumbens; VC_R, right vision cortex.

In the analyses of brain volumes in relation to hearing loss or tinnitus, we included 5222 individuals, among whom 311 had hearing loss, 720 had tinnitus and 117 had both. Hearing loss was associated with a smaller brain volume in all ROIs, although a statistically significant result (FDR < 0.05) was observed only for ten ROIs, including whole intracranial volume, whole grey matter volume, and volumes of bilateral cingulate gyrus, hippocampus, inferior temporal gyrus, parahippocampal gyrus, postcentral gyrus, precentral gyrus, right lateral grey matter hippocampus, and left lateral central opercular cortex and occipital pole (Fig. 3). In the inferior temporal gyrus and cingulate gyrus, a smaller brain volume in relation to hearing loss was mainly observed in the posterior division, whereas in the parahippocampal gyrus, a smaller brain volume in relation to hearing loss was primarily observed in the anterior division. Tinnitus was associated with a greater brain volume in the left accumbens. Having both hearing loss and tinnitus showed no statistically significant association with brain volume in any of the ROIs, perhaps owing to the small number of participants with both hearing loss and tinnitus. After adjustment for whole intracranial volume, there was no statistically significant association between hearing loss or tinnitus and the volume of any of the ROIs, apart from the positive association noted between tinnitus and greater volume of the left accumbens and right occipital pole (Fig. 4).

## Discussion

In the largest community-based cohort study to date based on the UK Biobank, we found that hearing loss and tinnitus were associated with an increased subsequent risk of depression and anxiety. We further found that hearing loss was associated with a smaller volume in the majority of the tested brain regions, whereas tinnitus was associated with a greater volume of the left accumbens. The former associations disappeared after further adjustment for whole intracranial volume, whereas tinnitus was found to be associated with greater volume of the left accumbens and the right occipital pole even after adjustment for whole intracranial volume.

### Depression and anxiety

The positive associations of hearing loss and tinnitus with risk of depression and anxiety are in line with findings of previous studies with either cross-sectional or longitudinal designs.^5,6,8,23,24^ Our results extend previous knowledge by showing that the associations were independent of potential confounders, including sociodemographic factors, history of other psychiatric disorders and insomnia; by disentangling the impact of hearing loss alone, tinnitus alone and the presence of both; and by demonstrating a temporal trend of the associations over time, as well as a dose–response relationship by severity of hearing loss and tinnitus.

In the present study, hearing loss was associated with a slightly increased risk of depression and anxiety, and the magnitude of the risk increment increased over time during the follow-up. In the secondary analyses, we further found that ‘poor hearing’ was more strongly associated with the risk of depression and anxiety than was ‘insufficient hearing’. This dose–response relationship adds further evidence to a potentially real association between hearing loss and mood disorders. Various reasons might underlie these results. For instance, negative emotional experiences related to hearing loss, such as loneliness or social isolation, might contribute to the increased risk of subsequent mood disorders.^25–27^ Besides, hearing impairment might lead to deafferentation-induced atrophy in auditory–cognitive control networks by reducing peripheral auditory input, which could also increase risk of depression and anxiety.^28,29^

Tinnitus was also associated with an increased risk of depression and anxiety. The relative risk seemed to be rather stable over time and demonstrated no clear temporal pattern. Also, a dose–response relationship was noted by severity of tinnitus, i.e. ‘bothersome’ versus ‘not bothersome’. Similar to those with hearing loss, individuals with bothersome tinnitus might feel especially annoyed and upset because of the noise in their ears, leading to negative emotional experiences^2,30^ and development of mental illness,^1,31,32^ whereas individuals with not-bothersome tinnitus might have adapted to the tinnitus situation better, although the latter are still at higher risk of depression and anxiety compared with individuals with neither hearing loss nor tinnitus.

### Brain MRI

In the present study we examined brain volume through a whole-brain explorative approach including cortical and subcortical regions (Supplementary Fig. 3). After adjustment for known confounders such as sex, age and other covariates,^10,18,20,33^ hearing loss was found to be associated with smaller brain volume in all studied ROIs, including the whole intracranial volume and whole grey matter volume, as well as the volumes of the auditory cortex and limbic system and their connective regions, and of the motor cortex, sensory cortex and vision cortex. This finding corroborates previous studies suggesting that hearing loss might be an indicator of a generic accelerated brain atrophy; it also extends previous knowledge by showing more affected regions.^34,35^ Indeed, a growing number of studies suggest that hearing impairment is associated with various neurodegenerative diseases, including cognitive impairment and dementia.^36,37^ The fact that the associations between hearing loss and smaller volume of different brain ROIs disappeared after further adjustment for whole intracranial volume suggests that hearing loss is not a biomarker for neuro-morphological changes in any specific brain region but rather a biomarker for such changes in the entire brain.

In contrast to hearing loss, tinnitus was not associated with the volume of the studied brain ROIs, apart from a greater volume in the left accumbens. Tinnitus also tended to be associated with a greater brain volume in the right accumbens, but the association was not statistically significant (data not shown). The fact that the positive association for the left accumbens persisted after further adjustment for whole intracranial volume suggests that the accumbens might be specifically involved in chronic tinnitus. In noise cancellation, the limbic system is a key area for the regulation of emotions and interacts with the auditory system, and the accumbens responds primarily to blocking signals that cause tinnitus perception to become chronic.^38,39^ However, owing to a lack of longitudinal MRI data, it remains unknown whether the greater accumbens volume is a pre-existing factor or a compensatory factor in chronic tinnitus.^12^ Tinnitus was also associated with a greater volume of the right occipital pole, after adjustment for the whole intracranial volume. This association is biologically plausible, as the primary visual cortex is located at the occipital pole, whereas an accurate orientation is based on the integration of auditory and visual information via white matter fibres connecting these regions.^40^ However, this is, to the best of our knowledge, the first study to report an association between tinnitus and grey matter of the occipital pole. More studies are therefore warranted to confirm this finding.

### Strengths and limitations

The strengths of the present study include the longitudinal study design based on a large community-based sample, the independently collected information on exposures (hearing loss and tinnitus) and outcomes (depression, anxiety and brain MRI data), and the objective measurements of hearing ability and severity of hearing loss using the DTT. Limitations of the study include the incomplete coverage of primary care and out-patient diagnosis of depression and anxiety, the lack of data on hearing thresholds and the relatively small number of individuals included in the analysis of brain MRI data. As we were primarily interested in new-onset depression and anxiety after the detection or self-report of hearing loss and tinnitus at recruitment to the UK Biobank, our study focused on a relatively old population without pre-existing depression or anxiety (mean age at recruitment: 57.4 years). It is therefore difficult to generalise our findings to the general population. In addition, as we had few data on the use of hearing aids or tinnitus maskers, further studies are needed to understand the mitigating effect of such treatments on the risk of mood disorders or neuro-morphological changes.

In conclusion, we found that hearing loss and tinnitus were both associated with a higher risk of depression and anxiety in a dose–response manner. Whereas hearing loss was associated with a smaller volume of certain brain regions, tinnitus was associated with a greater volume of the accumbens. As a result, hearing loss might be a risk factor for both mood disorders and altered brain morphology, whereas tinnitus might be more closely related to mood disorders.

## Supporting information

Chen et al. supplementary material Chen et al. supplementary material

## Data Availability

Data used in the present study can be requested from the UK Biobank. Copyright © 2023, NHS England. Re-used with the permission of the UK Biobank. All rights reserved.
