# Supplementary material for: Depression, anxiety and brain volume after hearing loss and tinnitus: cohort study in the UK Biobank
Source: BJPsych Open. 2024 Feb 1;10(2):e37. doi: 10.1192/bjo.2023.634 (PMC10897703; doi:10.1192/bjo.2023.634)

Supplementary Methods

**Digit Triplet Test**

Digit Triplet Test (DTT) is a speech-in-noise test for large population hearing screening, which has been described in detail previously (<http://biobank.ctsu.ox.ac.uk/crystal/label.cgi?id=100049>). In brief, 15 sets of three monosyllabic digits (e.g., 1-5-8) are presented via surround headphones. The ears are tested separately with randomized testing order among participants. In the beginning, participants set the volume of the stimuli to a comfortable level. Digit triplets are then presented in a background of noise shaped to match the spectrum of the speech stimuli. Noise levels vary adaptively after each triplet to estimate the signal to noise ratio (SNR) for 50% correct recognition of the three digits via touchscreen response. The recognition threshold is taken as the mean SNR for the last eight triplets. In the present study, hearing ability was determined based on performance of the “better-ear” and categorized according to the mean speech reception threshold (SRT) as “Normal” (SRT <−5.5 dB), “Insufficient” (SRT −5.5 to −3.5 dB), and “Poor” (SRT >−3.5 dB).

**Table S1. ICD-10 codes used to ascertain depression, anxiety, and previous psychiatric disorders**

| Psychiatric disorders | ICD-10 codes |
| --- | --- |
| Depression | F32, F33 |
| Anxiety | F40, F41 |
| Previous psychiatric disorders | F10-F19, F23-F25, F28-F34, F38-F45, F48,  F50-55, F59-F69, F81-F83, F88-F95, F98, F99 |

**Table S2.** **Hazard ratio (HR) and 95% confidence interval (CI) of depression or anxiety in relation to severity of hearing loss, using no hearing loss as reference***

|  | HR (95%CI) of depression | | | HR (95%CI) of anxiety | |
| --- | --- | --- | --- | --- | --- |
| Group | Insufficient hearing | Poor hearing |  | Insufficient hearing | Poor hearing |
| Overall | 1.08 (0.99-1.17) | 1.34 (1.14-1.57) |  | 1.10 (1.01-1.19) | 1.23 (1.04-1.45) |
| Stratified by sex |  |  |  |  |  |
| Male | 1.07 (0.94-1.22) | 1.23 (0.96-1.56) |  | 1.10 (0.96-1.26) | 1.23 (0.96-1.59) |
| Female | 1.08 (0.97-1.20) | 1.43 (1.15-1.76) |  | 1.09 (0.99-1.20) | 1.23 (0.99-1.52) |
| Stratified by previous psychiatric disorders | |  |  |  |  |
| Yes | 0.99 (0.8-1.24) | 1.42 (1.00-2.03) |  | 1.00 (0.80-1.24) | 1.04 (0.68-1.59) |
| No | 1.09 (1.00-1.19) | 1.32 (1.10-1.57) |  | 1.10 (0.96-1.26) | 1.23 (0.96-1.59) |
| Stratified by insomnia |  |  |  |  |  |
| Usually | 1.05 (0.93-1.20) | 1.27 (0.98-1.64) |  | 1.21 (1.07-1.37) | 1.20 (0.91-1.57) |
| Sometimes | 1.13 (1.00-1.28) | 1.40 (1.11-1.76) |  | 1.04 (0.93-1.17) | 1.30 (1.03-1.64) |
| Never | 0.98 (0.79-1.22) | 1.34 (0.89-2.01) |  | 0.98 (0.80-1.21) | 1.13 (0.73-1.74) |

*Adjusted for age, sex, income, educational level, insomnia, Townsend index, previous psychiatric disorders, and assessment center. All analyses used a 90-day lag time.

**Table S3.** **Hazard ratio (HR) and 95% confidence interval (CI) of depression or anxiety in relation to severity of tinnitus, using no tinnitus as reference***

|  | | HR (95%CI) for depression | | | HR (95%CI) for anxiety | | | |
| --- | --- | --- | --- | --- | --- | --- | --- | --- |
| Group | | Not-bothersome | Bothersome |  | Not-bothersome | | Bothersome | |
| Overall | 1.19 (1.10-1.28) | | 1.60 (1.43-1.78) |  | 1.21 (1.12-1.30) | | 1.71 (1.53-1.90) | |
| Stratified by sex | |  |  |  |  | |  | |
| Male | | 1.15 (1.03-1.30) | 1.59 (1.34-1.88) |  | 1.16 (1.03-1.31) | | 1.75 (1.47-2.08) | |
| Female | | 1.21 (1.09-1.34) | 1.59 (1.37-1.85) |  | 1.24 (1.13-1.37) | | 1.68 (1.46-1.93) | |
| Stratified by previous psychiatric disorders | | | | |  |  | |  |
| Yes | | 1.40 (1.17-1.68) | 1.57 (1.20-2.04) |  | 1.29 (1.06-1.57) | | 1.36 (1.01-1.83) | |
| No | | 1.15 (1.05-1.25) | 1.60 (1.42-1.81) |  | 1.20 (1.10-1.30) | | 1.78 (1.59-2.00) | |
| Stratified by insomnia | | |  |  |  | |  | |
| Usually | | 1.28 (1.14-1.44) | 1.66 (1.42-1.94) |  | 1.32 (1.17-1.48) | | 1.66 (1.41-1.95) | |
| Sometimes | | 1.12 (1.00-1.26) | 1.51 (1.27-1.80) |  | 1.19 (1.06-1.33) | | 1.74 (1.47-2.05) | |
| Never | | 1.06 (0.86-1.31) | 1.66 (1.15-2.39) |  | 0.96 (0.78-1.19) | | 1.75 (1.25-2.46) | |

*Adjusted for age, sex, income, educational level, insomnia, Townsend index, previous psychiatric disorders, and assessment center. All analyses used a 90-day lag time.


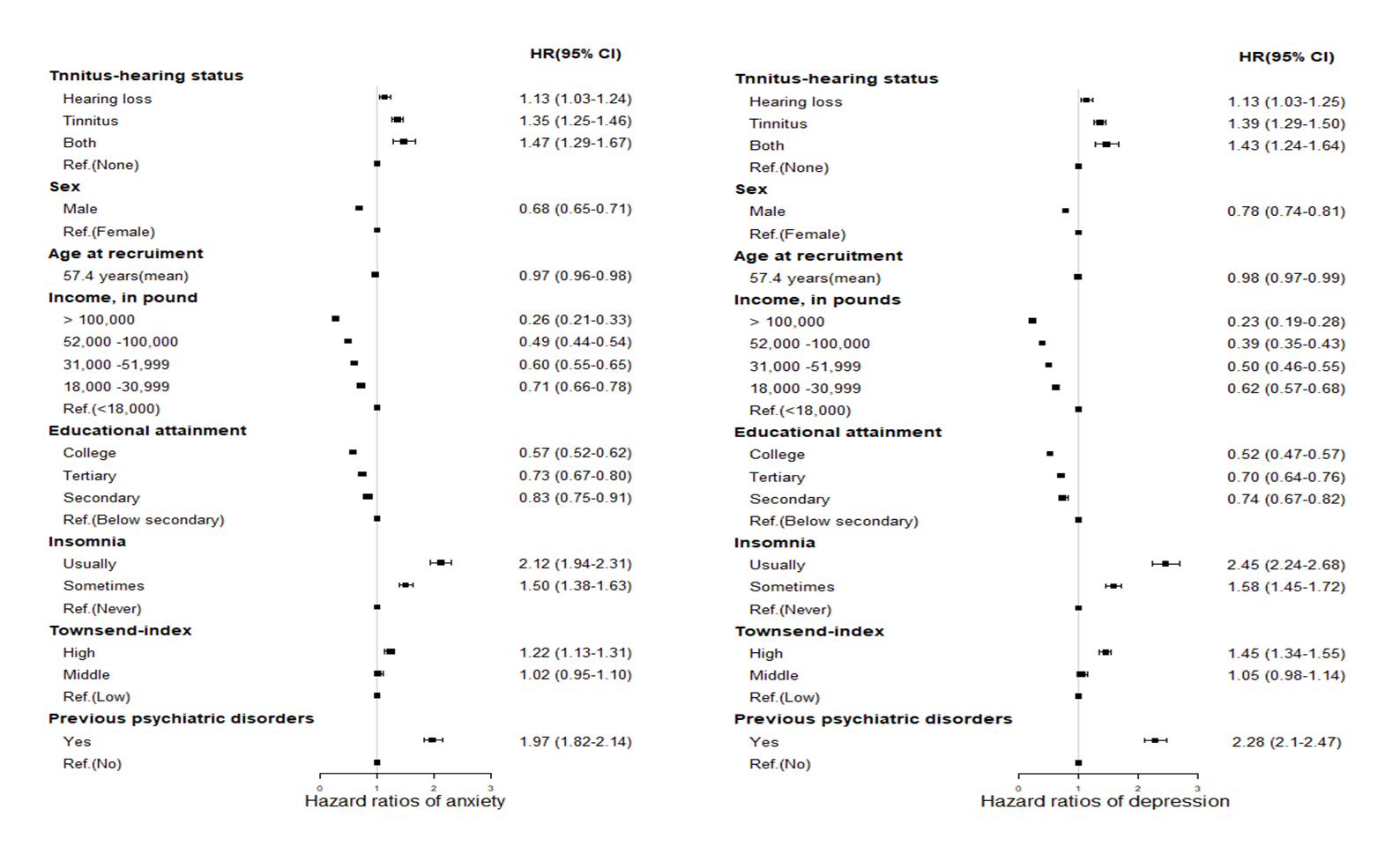
Figure S1. Hazard ratio (95% confidence interval) for depression and anxiety in relation to hearing loss or tinnitus and covariates, univariable analysis*

*All analyses used a 90-day lag time.

Figure S2. Changes of hazard ratios for depression and anxiety in relation to severity of hearing loss and tinnitus during the follow-up period


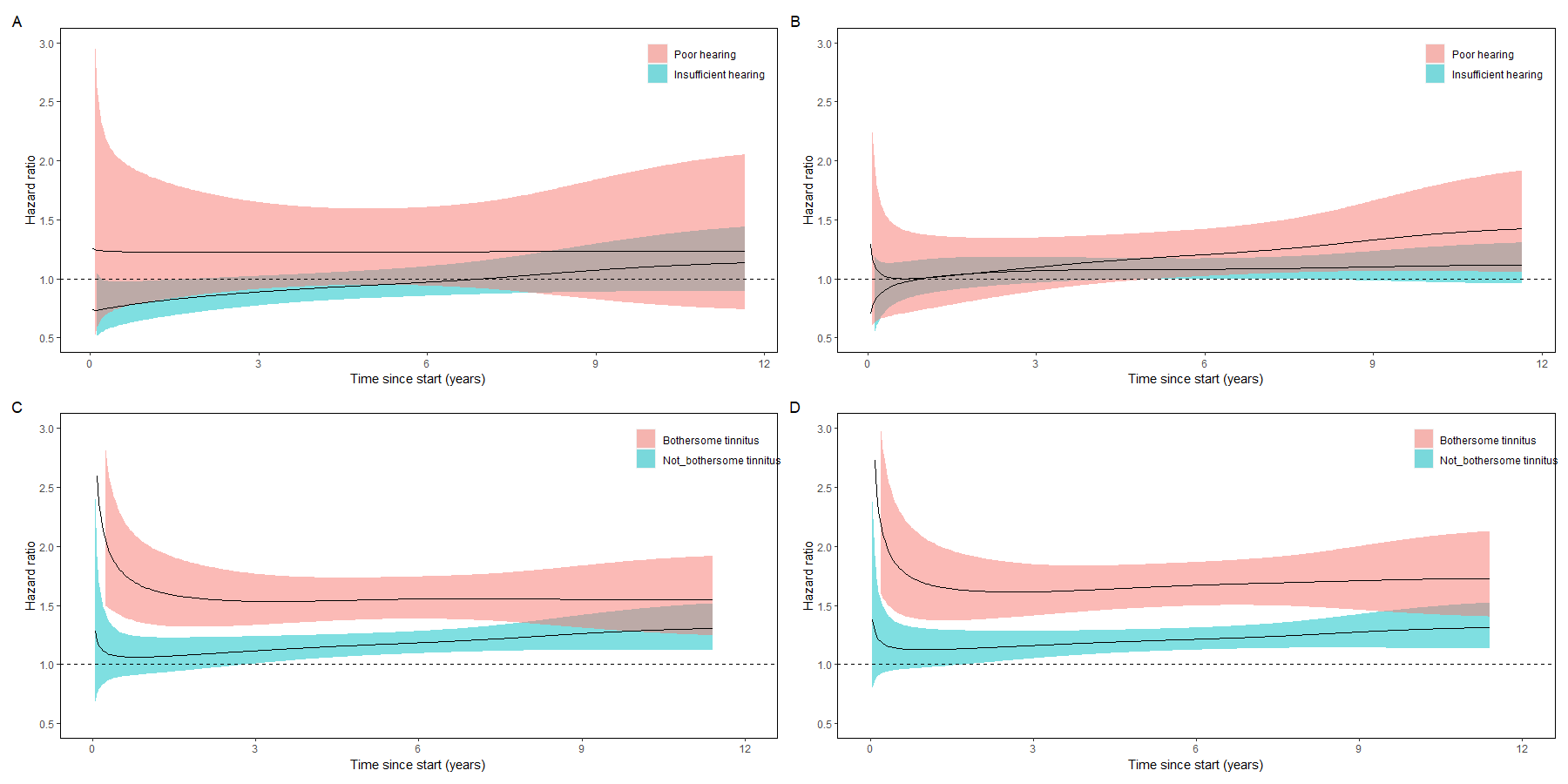


Flexible parametric model was used to demonstrate the changes of hazard ratios along the follow-up period, with neither hearing loss nor tinnitus as reference. All analyses used a 90-day lag time. A/C, depression. B/D, anxiety.

Figure S3. Structures of limbic system


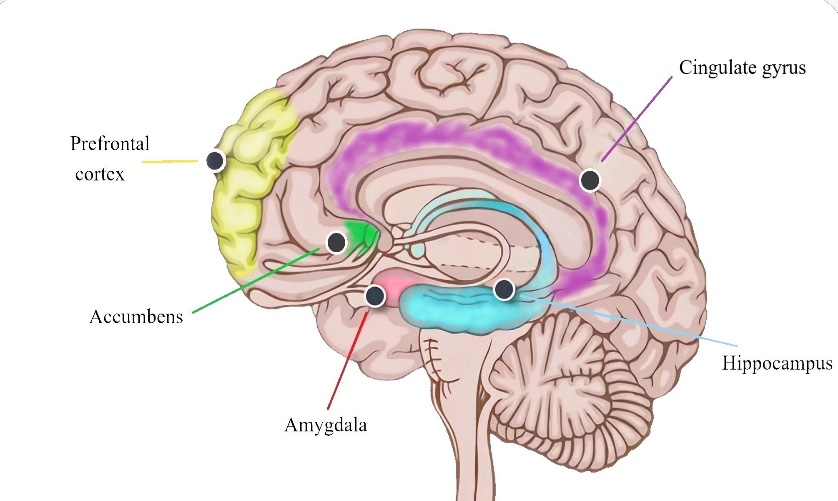

Supplement: Chen et al. supplementary material [file S2056472423006348sup001.docx]
